# A Framework for the Implementation of Digital Mental Health Interventions: The Importance of Feasibility and Acceptability Research

**DOI:** 10.7759/cureus.29329

**Published:** 2022-09-19

**Authors:** Susanna Y Park, Chloe Nicksic Sigmon, Debra Boeldt

**Affiliations:** 1 National Mental Health Innovation Center, University of Colorado Anschutz Medical Campus, Aurora, USA

**Keywords:** digital mental health, mental health technology, implementation, acceptability, feasibility, mental health

## Abstract

Digital mental health interventions (DMHIs) have the potential to serve a significantly wider portion of the population in need of mental health services. The coronavirus disease 2019 (COVID-19) pandemic has especially highlighted the exacerbation of mental health disparities among minoritized populations. Innovations and research on DMHIs continue to expand, reinforcing the need for a more systemic process of DMHI implementation. In practice, DMHI implementation often skips the fundamental steps of conducting acceptability and feasibility studies. We propose a DMHI implementation framework that identifies an acceptability and feasibility study as an essential first step, simultaneously centering equitable processes that address populations disproportionately affected by mental illness.

## Introduction and background

Digital mental health interventions (DMHIs) are technology-based interventions in the form of mobile apps, web-based programs, virtual reality (VR), wearable devices, or video games [1,2]. The technology can be used in the mental health space for prevention, education, assessment, and treatment. Since 2020, the coronavirus disease 2019 (COVID-19) pandemic catalyzed the need for innovative mental health services and resources as the demand for mental health care increased [3]. The subsequent lack of such services moved digital mental health to the forefront of addressing this gap [1], and funding increased significantly to accelerate the implementation of the technology. Telehealth use skyrocketed since the onset of the COVID-19 pandemic but mainly among persons already utilizing mental health services [4]. Leveraging technology using feasible and acceptable implementation methods represents a promising strategy to increase the engagement and utilization of mental health resources among minoritized racial groups, low-income individuals, and persons identifying as part of other underserved groups [5]. Applications that provide easy access to mental health support are an important method for promoting engagement in care, especially given that nearly 100% of individuals who identify with these groups have mobile devices, and over 80% use smartphones [6].

While DMHIs have the potential to reach a wider population than traditional mental healthcare and significantly increase access to mental health care, interventions must be developed systematically. Acceptability and feasibility studies are crucial first steps, and researchers must have a clear understanding of what acceptability and feasibility mean as measurable outcomes [7]. 

Acceptability

This is a patient’s or user’s willingness to use the DMHI. Researchers aim to understand the factors that increase use and engagement. We also want to understand why users disconnect from the product or the barriers to using the DMHI. These factors can be assessed via focus groups, interviews, structured questionnaires, and backend data.

Feasibility

The degree to which the technology or product can be successfully integrated within the flow of usual care. This is an iterative process often measured by the frequency of DMHI use. Researchers must identify the patient, provider, user, and operational barriers to DMHI use. 

In our framework, acceptability determines whether the community needs mental health services and whether the DMHI can meet these needs. Feasibility determines whether the users, administrators, and developers of the DMHI are satisfied [8]. Overlooking these factors can lead to unnecessary budgetary costs and inaccurate assessments of the effectiveness of the DMHI in clinical settings and use cases of general wellness support, thereby impacting the long-term sustainability and impact [8-11]. The ultimate goal to provide support for the efficacy and effectiveness of the DMHI requires the ability to implement and scale the digital mental health product in a larger population setting and measure its effectiveness according to the developed measures that were iteratively conceived and refined from the acceptability and feasibility processes. As digital mental health researchers, we aim to discuss the value of acceptability and feasibility studies and how we have applied them in real-world settings.

## Review

Research design suggestions

Acceptability and feasibility studies are fundamental to creating evidence-based and equitable digital mental health interventions. Researchers must be able to distinguish between whether a proposed DMHI is possible and whether it “works.” The second implies efficacy and requires a hypothesis [7,12], but this cannot be tested accurately if the intervention itself is not being implemented appropriately and to its full capacity. Pilot studies that assess the acceptability and feasibility of a DMHI provide valuable information to refine the intervention prior to an efficacy trial. At the pilot stage, we determine whether the intervention is needed and possible (acceptability) and whether all parties involved are satisfied with the intervention’s integration within the flow of the user’s usual care or routine (feasibility) [7,8].

**Figure 1:**
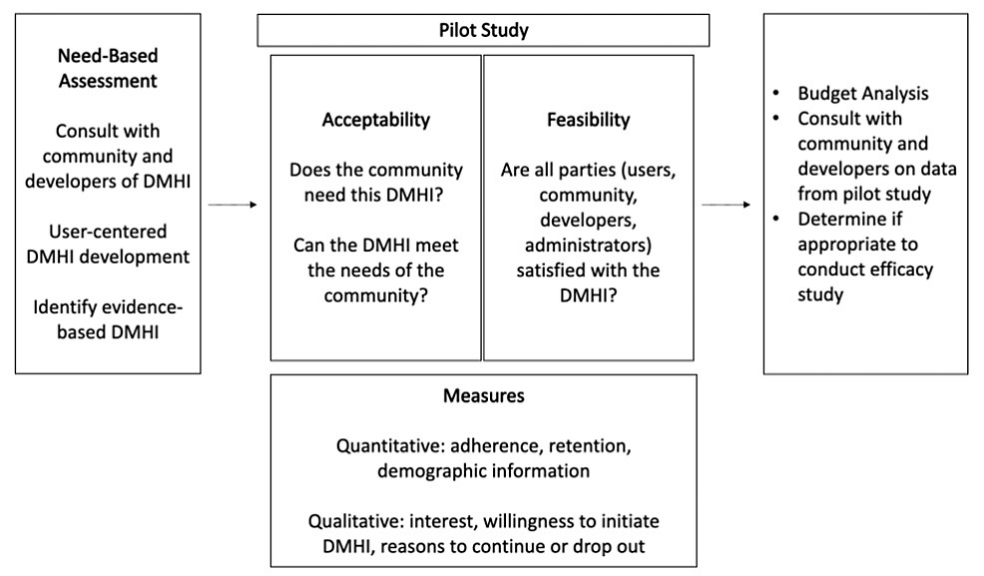
Framework for Implementation of Digital Mental Health Interventions

The proposed implementation framework (Figure 1) emphasizes conducting a needs assessment with organizations to strengthen the acceptability and feasibility of the intervention. Understanding the specific and unique needs of an organization or implementation setting allows researchers or organization leaders to gather comprehensive data on user perspectives on the DMHI and suggest data-driven improvements to the developers. As implementation researchers, we conduct pilot studies using DMHI products from technology companies and test them in real-world settings. The goal of our work is to implement DMHIs in a meaningful way; the DMHI must be evidence-based and receptive to change, and the change should be supported by appropriate facilitation [13]. If a pilot study is indicated, the first step to ensure a successful systemic process toward implementation is to conduct a pilot study that adequately addresses acceptability and feasibility. Examining the acceptability and feasibility of a DMHI can prevent having to compromise the quality of the intervention and can enable the ability to scale the intervention for the intended population appropriately. Acceptability and feasibility studies further strengthen the sustainability and scalability of the DMHI implementation, as these require measurements to be developed iteratively and according to the feedback obtained from the pilot study population. Such iterative processes allow us to answer whether the DMHI is possible through developed measures. For example, we may obtain quantitative data on engagement rates (e.g., adherence) and qualitative data on how likely the individual, organization, community, or clinician will use it in their practice [13]. While our research and knowledge on the DMHI are valuable, implementation of the DMHI may differ according to the specific needs of the community partner. Through collaboratively working with a community partner and technology company, back-end data can be tracked, such as adherence rates and specific areas of engagement within the technology, without burdening the community sites to collect these data.

We utilize a mixed-methods approach by collecting both quantitative and qualitative data, which enables a more comprehensive evaluation of the DMHI according to the unique needs of the population such as changes that need to be made to the content of the intervention, overcoming any operational barriers, and assessing budgetary boundaries. Both qualitative data, such as focus groups and interviews, and quantitative data, such as structured questionnaires, can be used to understand why or why not someone is inclined to engage with the intervention. Identifying barriers early also allows developers to workshop solutions and refine technical or operational challenges (i.e., feasibility) [7]. Quantitative data obtained during the pilot study can also be used to assess feasibility such as frequency of use and the adherence rate to the DMHI. A randomized design is not required at this stage, and no inferential statistics are applied. The “participant flow, budgetary constraints, and the number of participants needed to reasonably evaluate feasibility goals” are the primary concerns [12]. Safety and tolerability cannot be assessed at this stage, and the effect size of power calculations is unstable due to the nature of the study and the small sample size [12]. Critical nuances are highlighted through qualitative data, such as reasons for dropouts and perceptions of the usefulness of the DMHI according to their needs. Qualitative feedback gathered from the community sites is communicated with the developers of the DMHI so appropriate adjustments can be made. If our measures indicate high acceptability and feasibility rates, we can then consider moving toward an efficacy study.

Common pitfalls and mistakes

Conducting an Efficacy Study

One of the most common mistakes in DMHI implementation is the failure to adequately determine the acceptability and feasibility of the intervention prior to conducting efficacy trials. Efficacy trials are an essential step in identifying evidence-based DMH but they should be conducted after a feasibility/acceptability pilot demonstrates adequate engagement and retention. Researchers will often overlook whether the DMHI is possible (e.g., assuming that since the content is evidence-based in other delivery formats, implementation will be the same as in typical settings) and attempt to answer whether the intervention “works.” In doing so, they gather “preliminary efficacy” data, which do not provide adequate information on the safety and usability of the DMHI [7,12]. Pilot studies that correctly examine acceptability and feasibility create room for improved iterations of the DMHI so that it can be more readily applied in an efficacy study. Pilot studies that forego acceptability/feasibility and attempt to determine “preliminary efficacy” can result in a type II error, or false negative result, in which the analysis reveals a small effect size. This has unwarranted and detrimental ripple effects on the future of the DMHI, such as not pursuing an efficacy study, difficulty funding the study, or requiring an unnecessarily large number of participants. On the other hand, we may encounter a type I false positive, in which the study reveals a strong effect size. This has a ripple effect in which the following trial is underpowered, therefore failing to detect any clinically meaningful effect [12]. Therefore, evaluating feasibility and acceptability is imperative to accurately advance existing knowledge of DMHI implementation and outcomes. 

Failing to Consider Digital Equity

As DMHIs continue to grow, developers and researchers alike must address the inequities that continue to exist in mental health and well-being among populations. Prior to the COVID-19 pandemic, we saw disparities among people who were of minoritized racial/ethnic backgrounds, low socioeconomic status, older, living in rural settings, identifying as LGBTQ+, and/or having a disability. Access to quality mental health care services becomes even more urgent for these populations, as the COVID-19 pandemic added additional challenges to well-being. DMHIs have the potential to narrow this gap if they are developed and implemented with an equity-based framework.

Inequities in digital mental healthcare are not simply due to a lack of access. Acceptability and feasibility studies are opportunities for us to explore beyond what the individuals, communities, and population lack. Instead, we can determine systemic reasons for the lack of engagement with the DMHI, elevate their existing connections and understandings of technology, and provide sustainable educational opportunities to increase digital health literacy. Without the intentional inclusion of those who are already disproportionately affected, digital mental health services will continue to serve those already engaged and increase pre-existing inequities. 

Inequities in the digital healthcare space are evident in the digital divide. The National Digital Inclusion Alliance defines the digital divide as the gap between those who are able to engage online and those who cannot due to a lack of access, skills, and/or support [14]. The digital divide is most likely to impact those persons and groups who already face disparities in traditional mental health care. For instance, Americans with a disability are less likely to have a traditional computer or a smartphone compared to those without a disability [15]. Further, low-income adults are more likely to be smartphone-dependent, rely solely on their smartphones, and rely less on other technological devices than those of higher socioeconomic status [16]. Financial barriers also contribute to the lack of home broadband internet access among low-income households and those who live in rural areas [16-18].

Inequities also exist beyond the digital divide. The rapid development and deployment of DMHIs often rely on sustained connectivity, English language proficiency, and sufficient digital literacy. Utilization of mental health care services among people of color is lower than their white counterparts, but research thus far shows that people of color are as willing or more likely to engage with DMHIs than their white counterparts [19,20]. Developers and researchers who conduct acceptability and feasibility studies with specific marginalized populations in mind can identify cultural needs and barriers that can be addressed early on. This work is critical in order to be able to make clinical validation claims for these populations. As DMHI research continues to expand, we observe a lack of non-white user representation in data for both the technology and the research assessing the technology, which can significantly impair the ability for accurate generalizable conclusions. Centering the voices of those who are underrepresented in the development and research stages is an essential first step to facilitating using the DMHI by such groups. 

Acceptability and feasibility in practice

As described above, our team has learned many lessons as we have gained experience and expertise in implementing digital mental health. We have worked collaboratively with a wide variety of community partners (e.g., K-12 schools, universities, corrections, workplace wellness for business organizations, mental health clinics, and hospitals) to deploy VR and other types of technology focused on mental health and wellness (e.g., smartphone apps, wearables, etc.). We will review our experience deploying a DMHI in a residential substance use facility as a case study and share the insights gained.

Implementation considerations in residential substance use disorder (SUD) programs include employees at all levels (i.e., leadership, clinicians/mental health providers, and support staff) as well as patients. During our needs assessment, we discovered that the site was interested in innovative recovery support tools and vetted various DMHIs to find evidence-informed VR mindfulness content that fits the site’s needs and could be implemented within their existing programming. Obtaining buy-in from facility leadership was critical for problem-solving feasibility barriers related to deployment and initial implementation. Having support from leadership facilitated our team’s ability to conduct training and work with staff to understand the benefits of implementing DMHIs for persons with substance use disorders both during and after treatment in a residential setting. In some instances, physicians' lack of time makes it infeasible to train clinicians on the technology. In these cases, the support staff has taken on a wide variety of responsibilities essential for sustainable implementation (e.g., charging and updating software on equipment, tracking down patients, identifying times within patient treatment schedules when they are available to engage with the technology, and providing technical support). We have learned that adequate employee orientation and training, along with a point of contact for ongoing technical support, significantly increases both technology use and staff and patient's perceptions of the technology's usefulness. With regard to feasibility at the patient level, we benefitted from taking the time to thoroughly plan specific implementation details. For example, creating time within patients’ existing clinical schedules during which they can realistically engage with the technology is essential and is something that will require an ongoing assessment to understand and problem-solve barriers to use. Furthermore, the assumption that patients will all feel similarly about their mastery and willingness to use technology is faulty. Understanding, prior to the implementation, how patients feel generally about technology use can help inform decisions such as time spent on initial training and methods for orienting patients to the tool (e.g., focus on evidence supporting effectiveness and methods for making engagement user-friendly and appealing). We have received overwhelmingly positive feedback from residential SUD patients about VR mindfulness when they have been fully oriented and trained on how to use the equipment appropriately (e.g., adjusting the headset so the content is not blurry, navigating access to multiple types of content) and when they have been given time within their schedule to engage. We have learned that as a result of this training process, staff and providers, in addition to facilitating patient use, are often interested in using the DMHI to support their own wellness.

One overarching factor was relevant across all settings in which we deployed mental health technology: how equity is addressed within the setting impacts technology engagement. Our team initially focused our efforts on deploying DMHI to local community mental health centers since they are often the last to get access to or adopt innovative treatment methods. In general, we target implementation to underserved populations who are most likely to encounter access barriers to traditional mental healthcare or other wellness support. Feasibility factors are addressed first, including working with the organization’s leadership and administration to understand their needs and developing a plan for deployment that fits into already existing systems and training technology. We then put equal effort into ensuring acceptability by assessing what factors individual users like and do not like and how these factors contribute to their willingness to repeatedly engage with the DMHI. We have found that successful deployment within underserved settings allows persons in these settings to access high-quality, evidence-informed mental health interventions and/or wellness tools that may have otherwise been inaccessible. As we continue our implementation efforts, we continue to be mindful of how the DMHI we utilize may be influencing equity. For example, much of the technology that we deploy can be accessed through a smartphone, a technology to which most people in the US have access [6]. Alternatively, when we deploy DMHI using VR, which is not often feasible for users to purchase for individual use, we focus on how we can help the organization to facilitate access to individuals within the organization in a sustainable way (e.g., as part of a treatment program, during school hours, etc.).

Recommendations

As a result of our diverse experiences deploying DMHIs using methods that are both sustainable and scalable over time, we have developed recommendations for achieving feasible and acceptable implementation. These recommendations can be considered trans-organizational and customizable, applying to most settings in one way or another.

Select the DMHI That Fits the Organizations' and Users' Needs

It is critical to understand the pain points of both the organization at which the DMHI will be deployed and the individuals who will use the DMHI. Technology has the potential to transform the delivery of mental healthcare and wellness services, but only if leveraged appropriately. When selecting a DMHI, consider carefully what targets need to be addressed (e.g., reduced stress for employees) and how the DMHI will fit into existing structures (e.g., the technology available at work vs provided to employees to take home). If any of these organization-specific needs are ignored, feasibility and acceptability are both likely to take a hit and the sustainability of the implementation will suffer. 

Technology Should be Vetted Before Deployed

Not all DMHIs are created equal. The majority of what is available on the market has not been researched or clinically validated and does not utilize evidence-informed content. In the past decade, especially since the onset of the COVID-19 pandemic, technology development has skyrocketed. Funding to digital health startups grew 79% in 2021 with $57.2 billion put toward this sector [21]. A DMHI that is well-marketed does not mean it offers high-quality interventions, and there are different needs and standards for clinical use cases as opposed to DMHIs designed for stress management and wellbeing. Persons facilitating DMHI implementation for a particular setting should ensure that the specific technology selected has been vetted for quality. There is a range of characteristics of each DMHI that should be considered before beginning implementation. This includes factors specific to the individual and the organization more broadly [2].

Consider Customization When Possible

Individual engagement with DMHI is often higher when the content can be personalized to the user’s specific needs [22]. When vetting multiple DMHIs to make a final selection based on organizational needs and quality of content, it may be important to consider the ways in which they can be adapted for individual use. For example, can individuals select from multiple types of content (e.g., length of meditation, voice of guide) or track their progress (e.g., use, mood, etc.) over time? These factors may contribute to user acceptability and perception of DMHI effectiveness, and ultimately, may make the difference in whether implementation is sustained over time.

Choose DMHIs That Are Convenient, Easy to Use, and Anonymous

Users in any deployment setting are much more likely to engage with DMHI that is easily accessible, user-friendly (with easy access to technical support if needed), and gives the users confidence that their data (e.g., frequency of use data, responses, or data tracking within the content) will remain anonymous or confidential in that it will not be shared without their permission. Therefore, it is critical that organizations both research and understand what data are collected by the DMHI platform and exactly how those data will be used and then clearly and explicitly convey this information to users from organizations during the initial stages of implementation.

Orientation to and Framing of the Purpose of DMHI Matters

Users within an organization are more likely to use DMHI when it is positively framed and the benefits of use are clearly articulated. Depending on the setting, it may also be critical to address and remove any potential stigma associated with use through interventions such as leadership highlighting their own use or emphasizing that individual users will be completely anonymous. During the rollout of DMHI, explain why the intervention is being deployed, how it works, and how it fits with the bigger picture. Transparency and rationale during initial deployment set the stage for successful implementation. For example, highlighting that engagement with the DMHI is voluntary or explaining how any data collected will be used to support user well-being can help individuals believe the DMHI can be trusted and is safe to use. If a research study is part of the deployment, it is essential to share with individuals the purpose or aims of the study, how they are able to participate in gathering data, and how their data will be used/shared.

Facilitation of Training and Implementation

Successful implementation in specific settings requires that users know HOW to use the DMHI. Training approaches and methods should be customized and carefully planned, including the process, content, and availability of support. Digital navigators may be indicated for an organization to help guide these processes [23]. Further, implementation methods, such as the role of administrative support and how the rollout and/or impact of the DMHI will be evaluated, should also be determined via mindful consideration of what will be most sustainable for the organization [24]. Implementation frameworks have been developed for guidance around effectively hitting the balance between treatment adherence (i.e., matching implementation process to methods in published research demonstrating efficacy of a particular tool) and adaption of a specific DMHI to a particular setting. This allows for the implementation and sustained engagement with the DMHI to be feasible [2,13]. Further, much guidance around the development of qualitative data collection methods for further implementation has been published, including the importance of maximizing the use of established qualitative methods while also prioritizing situation-specific or organizational needs [25]. This body of literature can be practical and contains useful resources to support implementation efforts.

Program Evaluation, Quality Improvement, and Research Considerations

It is critical to assess the feasibility, acceptability, and effectiveness of deployment over time in order to maximize the likelihood that implementation will be sustainable. Organizations can do this as part of program evaluation or quality improvement efforts or can elect to run IRB-approved research pilots or comparison studies. Partnerships with academic institutions or experts in DMHI implementation research are often helpful in these efforts. These evaluations can help identify barriers to the feasibility and acceptability of DMHI implementation in the specific context, which will allow for problem-solving and the identification of concrete solutions that can be implemented. Semi-structured interviews are often a critical part of the evaluation; they shed light on what is driving successful implementation and what barriers are interfering with success. Objective engagement data (e.g., frequency of use) and standardized, validated assessments can also contribute to an in-depth understanding of the impact that the DMHI is having both at the organizational level and individual level. When research is conducted, implementation strategies should be reported as part of the publishing and dissemination process to help future dissemination efforts in other settings and to facilitate understanding of the implementation methods that resulted in DMHI effectiveness [26].

Significance of and potential for mental health technology to transform mental healthcare delivery

In a world of fast-paced development and ever-growing demand for mental health and wellness support, digital mental health interventions represent an ideal, and arguably essential, solution to address our current mental health crisis. As high-quality, evidence-informed content is paired with user-friendly, accessible, and transparent technology, DMHIs can make a significant impact. The implementation that directly addresses and overcomes feasibility and acceptability barriers is crucial for effective, sustainable, and scalable deployment. Organizational efforts aimed toward deployment in a way that facilitates individual use, willingness to engage, and perception of effectiveness will ultimately allow for equitable and effective mental healthcare delivery. 

Of note, related to the power to transform healthcare, we would be remiss to ignore the significant systemic shifts that would need to occur for DMHIs to be equitable for all populations. Part of DMHIs’ innovation is the ability to provide remote services while gathering patient data. In clinical use cases, these data can be transmitted to electronic healthcare record systems (EHR) to support measured clinical care [17]. Patient care equity can be achieved by promoting the interoperability of DMHIs and EHRs. This is particularly challenging in rural areas or places where they are slow to adopt EHR systems. However, this may be countered with reimbursement policies for DMHIs to incentivize the adoption of EHR systems [17]. There is another challenge regarding current state licensing requirements among mental health therapists, further restricting access to DMHIs as mental health professionals may not be able to operate in locations outside of their licensed state(s). In addition, billing codes and reimbursements are limited to licensed mental health professionals, narrowing the services that can be offered through DMHIs. Relaxation of such policies to include billing codes by non-mental health specialists, such as peer support and coaching services, can expand the type of services offered through DMHIs [17].

Limitations to DMHIs

The implementation of DMHIs requires multi-level assessment. The DMHI may be acceptable and feasible, but organizations or communities implementing the intervention may not see a valuable return on investment. There are valid concerns about potential negative outcomes associated with DMHIs. For example, research conducted since the onset of the COVID-19 pandemic demonstrates that in some instances, engagement with technology can negatively impact mental health such as increased use of video conferencing being linked to increased incidence of body dysmorphic disorder [27]. Another concern might be that digital mental health delivery platforms could encourage looser commitment to therapeutic relationships, which could affect attrition. Although these concerns can likely be mitigated by involving clinicians in driving outcome research for interventions, they are, nonetheless, critical to consider, systematically evaluate, and problem-solve [28]. Lastly, ensuring data privacy is a key factor in building trust with users, as there is a consistent threat against data privacy. Thus, it is imperative that developers, researchers, and organizations establish transparency in how the DMHI is developed, how the data are collected and used, and how the users experience the DMHI.

## Conclusions

There is a clear, urgent need for the rapid deployment of DMHIs to address the mental health needs of various populations. Technology companies and researchers who aim to implement DMHIs must do so with an evidence-based approach and equity framework. Our framework is developed using current research and retrospective insight from real-world applications. Too often, DMHIs are implemented without adequate acceptability and feasibility measures, which can be a detriment to all parties involved. Having a better understanding of how to conduct a pilot study to effectively evaluate acceptability and feasibility paves the way for safer, sustainable, and scalable solutions.

## References

[1] Khanna MS, Carper M (2022). Digital mental health interventions for child and adolescent anxiety. Cogn Behav Pract.

[2] Schueller SM, Torous J (2020). Scaling evidence-based treatments through digital mental health. Am Psychol.

[3] Torous J, Jän Myrick K, Rauseo-Ricupero N, Firth J (2020). Digital mental health and COVID- 19: using technology today to accelerate the curve on access and quality tomorrow. JMIR Ment Health.

[4] DuCharme J (2022). Teletherapy aimed to make mental health care more inclusive. The data show a different story. https://time.com/6071580/teletheraphy-mental-health/.

[5] (2022). Study of mental health treatment trends early in pandemic. https://about.kaiserpermanente.org/our-story/health-research/news/mental-health-treatment-rate-rose-early-in-pandemic.

[6] (2022). Mobile fact sheet. https://www.pewresearch.org/internet/fact-sheet/mobile/.

[7] Birckhead B, Khalil C, Liu X (2019). Recommendations for methodology of virtual reality clinical trials in health care by an international working group: iterative study. JMIR Ment Health.

[8] Myers KM, Valentine JM, Melzer SM (2007). Feasibility, acceptability, and sustainability of telepsychiatry for children and adolescents. Psychiatr Serv.

[9] Le LK, Esturas AC, Mihalopoulos C, Chiotelis O, Bucholc J, Chatterton ML, Engel L (2021). Cost-effectiveness evidence of mental health prevention and promotion interventions: a systematic review of economic evaluations. PLoS Med.

[10] Connolly SL, Hogan TP, Shimada SL, Miller CJ (2021). Leveraging implementation science to understand factors influencing sustained use of mental health apps: a narrative review. J Technol Behav Sci.

[11] Balcombe L, de Leo D (192021). Digital mental health amid COVID-19. MDPI.

[12] (2022). Pilot studies: common uses and misuses. https://www.nccih.nih.gov/grants/pilot-studies-common-uses-and-misuses.

[13] Ogden T, Fixsen DL (2014). Implementation science. A brief overview and a look ahead. Zeitschrift für Psychologie.

[14] (2022). The words behind our work: the source for definitions of digital inclusion terms. https://www.digitalinclusion.org/definitions/.

[15] Perrin A, Atske S (2022). Americans with disabilities less likely than those without to own some digital devices. https://www.pewresearch.org/fact-tank/2021/09/10/americans-with-disabilities-less-likely-than-those-without-to-own-some-digital-devices/.

[16] Vogels EA (2022). Digital divide persists even as Americans with lower incomes make gains in tech adoption. https://www.pewresearch.org/fact-tank/2021/06/22/digital-divide-persists-even-as-americans-with-lower-incomes-make-gains-in-tech-adoption/.

[17] Graham AK, Weissman RS, Mohr DC (2021). Resolving key barriers to advancing mental health equity in rural communities using digital mental health interventions. JAMA Health Forum.

[18] Vogels EA (2022). Some digital divides persist between rural, urban and suburban America. Some Digital Divides Persist between Rural, Urban and Suburban America.

[19] Friis-Healy EA, Nagy GA, Kollins SH (2021). It is time to REACT: opportunities for digital mental health apps to reduce mental health disparities in racially and ethnically minoritized groups. JMIR Ment Health.

[20] Kodish TA (2022). Digital mental health interventions for college students of color: understanding uptake and enhancing engagement. eScholarship.

[21] (2022). State of digital health 2021 report. https://www.cbinsights.com/research/report/digital-health-trends-2021/.

[22] Borghouts J, Eikey E, Mark G (2021). Barriers to and facilitators of user engagement with digital mental health interventions: systematic review. J Med Internet Res.

[23] Connolly SL, Kuhn E, Possemato K, Torous J (2021). Digital clinics and mobile technology implementation for mental health care. Curr Psychiatry Rep.

[24] Sullivan G, Blevins D, Kauth MR (2008). Translating clinical training into practice in complex mental health systems: toward opening the 'black box' of implementation. Implement Sci.

[25] Ramanadhan S, Revette AC, Lee RM, Aveling EL (2021). Pragmatic approaches to analyzing qualitative data for implementation science: an introduction. Implement Sci Commun.

[26] Rudd BN, Davis M, Beidas RS (2020). Integrating implementation science in clinical research to maximize public health impact: a call for the reporting and alignment of implementation strategy use with implementation outcomes in clinical research. Implement Sci.

[27] Sarangi A, Yadav S, Gude J, Amor W (2022). Video conferencing dysmorphia: assessment of pandemic-related body dysmorphia and implications for the post-lockdown era. Cureus.

[28] Aboujaoude E, Gega L (2019). From digital mental health interventions to digital “addiction”: where the two fields converge. Front Psychiatry.

